# Individual quality and phenology mediate the effect of radioactive contamination on body temperature in Chernobyl barn swallows

**DOI:** 10.1002/ece3.7742

**Published:** 2021-06-02

**Authors:** Zbyszek Boratyński, Timothy A. Mousseau, Anders Pape Møller

**Affiliations:** ^1^ CIBIO/InBio Research Centre in Biodiversity and Genetic Resources University of Porto Porto Portugal; ^2^ Department of Biological Sciences University of South Carolina Columbia SC USA; ^3^ SURA/LASSO/NASA ISS Utilization and Life Sciences Division Kennedy Space Center Cape Canaveral FL USA; ^4^ Ministry of Education Key Laboratory for Biodiversity Science and Ecological Engineering College of Life Sciences Beijing Normal University Beijing China; ^5^ Ecologie Systematique Evolution CNRS AgroParisTech Universite Paris‐Saclay Orsay France

**Keywords:** barn swallow, body condition, body temperature, *Hirundo rustica*, physiological performance, radiocontamination

## Abstract

Anthropogenic stressors, such as radioactive contaminants released from the Chernobyl and Fukushima Daiichi accidents, deteriorate ecological and evolutionary processes, as evidence for damaging effects of radioactive contamination on wildlife is accumulating. Yet little is known about physiological traits of animals inhabiting contaminated areas, and how those are affected by individual quality and phenology. We investigated variation in body temperature of wild barn swallows, *Hirundo rustica*, exposed to radioactive contamination from the Chernobyl accident in Ukraine and Belarus. We tested whether exposure to variable levels of radioactive contamination modified core body temperature of birds, and whether individual and phenological characteristics modulated radiosensitivity of body temperature. We showed that barn swallow body temperature varied with exposure to environmental radioactive contamination and that individual characteristics and phenology affected radioactive exposure. Increased radiosensitivity and up‐regulation of body temperature were detected in birds of low body condition, high risk of capture, and in animals captured late during the day but early during the season. These results highlight the complex ways that the body temperature of a wild bird is impacted by exposure to increased radioactive contamination in natural habitats. By impacting body temperature, increased radioactive contamination may compromise energetic balance, jeopardize responsiveness to global warming, and increase risk of overheating.

## Graphical abstract

Impaired regulation of core body temperature, along with the ongoing warming of climate, may compromise energetic balance and increase risk of extinction of populations and species that are living close to their energetically balanced physiological limits. Understanding the physiological effects of animal and human exposure to radioactive and other contaminates is an important issue given accumulating pollution in natural habitats.

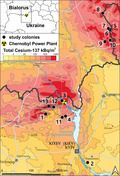

## 1 INTRODUCTION

Exposure to radioactive contamination can cause diverse problems for wildlife, such as decreased abundance of insects, impaired germination and development of plants, eye cataract in birds and mammals, or increased embryonic lethality and decreased reproduction in rodents (Cannon & Kiang, 2020). During the explosion and nuclear fire at the reactor in the Chernobyl power plant in 1986, a diversity of radioactive isotopes was released into the environment, and after more than three decades, they still influence ecosystems (Cannon & Kiang, 2020; Møller & Mousseau, 2006). The accidents at the Fukushima Daiichi power station, and studies that followed both disasters, reinforced awareness of the problems related to radioactive contamination (Steinhauser et al., 2014).

Consumption of contaminated water and food items and consequently ingestion of radionuclides such as cesium (^137^Cs), plutonium (^239^Pu), and strontium (^90^S), the main long‐term contaminants from the Chernobyl accident (Steinhauser et al., 2014; UNSCEAR, 2000), result in direct radioactive exposure to internal organs. Internal exposure can cause serious damage to cells by high‐energy *α* emitters (such as ^239^Pu), a source of radiation that normally does not penetrate the skin (Cannon & Kiang, 2020). Research has shown that animals absorb radionuclides from Chernobyl (Beaugelin‐Seiller et al., 2020; Beresford & Howard, 1991; Gashchak et al., 2008; Kalas et al., 1994; Map pes et al., 2019), and that radioactive contamination affects biology of diverse organisms (e.g., cataract in birds: Mousseau & Møller, 2013; fur color in voles: Boratyński et al., 2014; development in carrots: Boratyński et al., 2016; reproduction in voles: Lehmann et al., 2016; telomerase expression and telomere length: Kesäniemi, Lavrinienko, et al., 2019; gut microbiome: Lavrinienko et al., 2021). Among other effects, increased exposure to radioactive contamination can affect internal organs (e.g., reduced telomere length in the liver and testis: Kesäniemi, Lavrinienko, et al., 2019; reduced brain and kidney masses: Kivisaari et al., 2020; Møller et al., 2011). Damage to important organs can have serious consequences for animals' physiology, that can ultimately decrease fitness in Chernobyl animals (Ellegren et al., 1997; Lehmann et al., 2016). Yet very little is known about how animals experiencing ecologically relevant processes, such as competition for resources and foraging, physiologically respond to radioactive contaminants in their natural environment (Bonisoli‐Alquati et al., 2020; Garnier‐Laplace et al., 2013).

Endothermic physiology characterizes animals that maintain relatively stable and high body temperature by physiologically generated heat, thus investing more energy in thermoregulation than ectotherms (Angilletta et al., 2010; Legendre & Davesne, 2020; Ruben, 1995). Birds routinely maintain a stable body temperature of 37–40°C (Clarke & Rothery, 2008; McNab, 1966; Prinzinger et al., 1991), that can be even higher in aerial insectivores (i.e., birds capturing their prey at flight), such as barn swallows (Stoner, 1935). Accordingly, most birds are constantly exposed to a mild to moderate cold stress, investing substantial amounts of energy to thermoregulation. Birds and mammals have relatively high metabolic rates and daily energy expenditures compared to reptiles of the same size (Arnold et al., 2021). Any disruption to their energy budget can potentially be harmful and have consequences for fitness (Boratyński et al., 2010, 2013; Boratyński & Koteja, 2010). Exposure to a variety of toxic agents (e.g., methyl alcohol, benzene, paraldehyde, DDT, ozone) can hamper animals' normal physiological processes, but it is unclear how radioactive contamination affects thermoregulation (Gordon, 2018). Acute exposure to toxic chemicals (e.g., insecticides, metals) often involves initial down‐regulation of body temperature by physiological and behavioral heat loss. Such a response is usually followed by a delayed fever‐like elevation of core body temperature (e.g., after exposure to ethanol, organophosphates, or anticholinesterase: Gordon, 2010). In contrast to acute responses, mechanisms of delayed fever‐like responses after intoxication are not well understood (Bicego et al., 2007; Gordon, 2018). Yet, fever can be sustained for up to 4 days after acute alcohol intoxication in rats (Gordon, 2010). Fever has also been detected as chronic response (e.g., in timber industry workers exposed to pentachlorophenols used as antifungal wood treatment; Gordon, 2010), and fever can significantly affect animals' behavior and fitness (Adelman et al., 2010; Kluger et al., 1975; Sauer et al., 2019). It is expected that animals in Chernobyl are exposure to low‐dose but long‐term and chronic radioactive contamination. Therefore, if thermoregulatory consequences of exposure to radioactive contamination are similar to those caused by toxic chemicals, development of fever can also be predicted.

**FIGURE 1:**
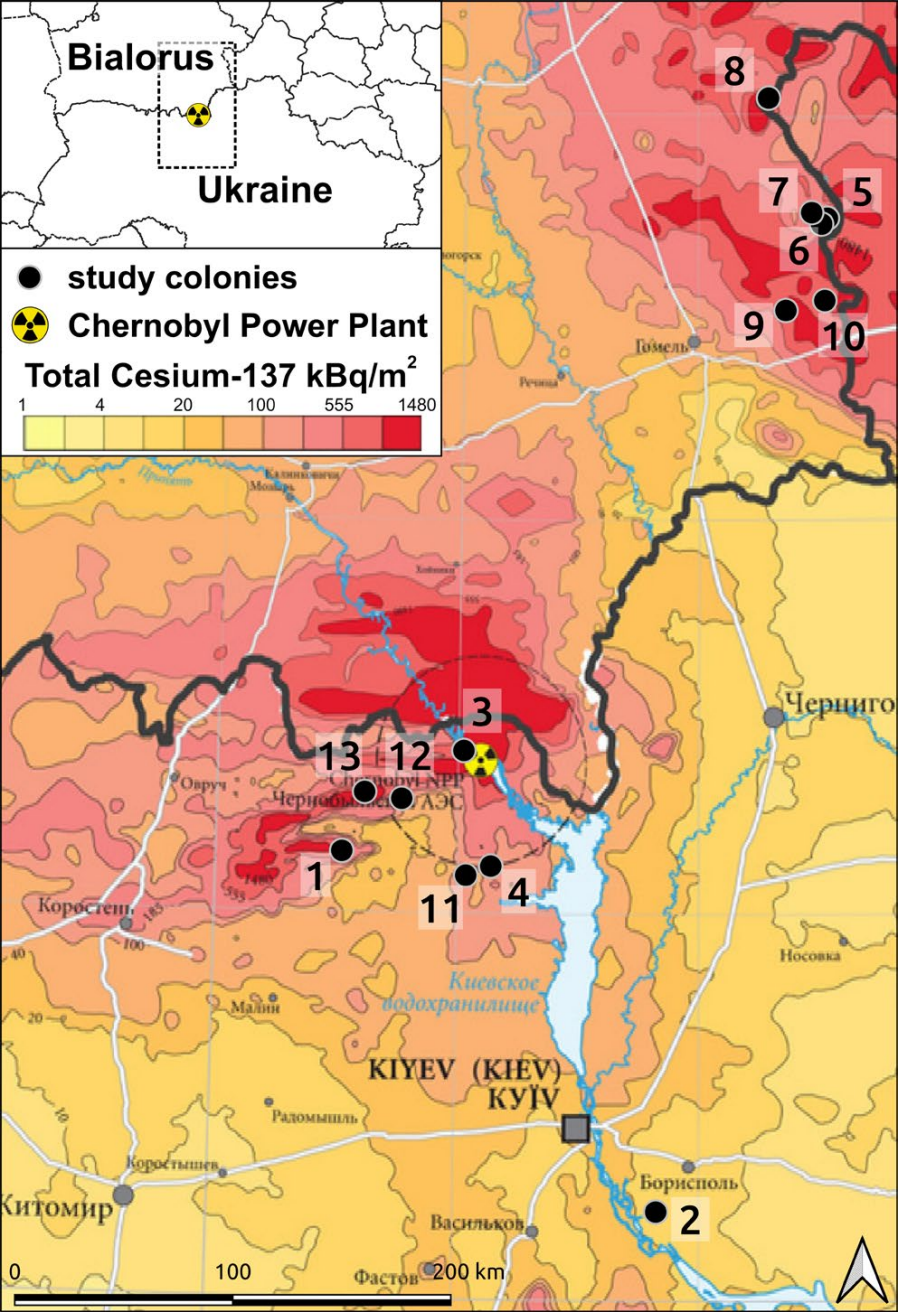
Spatial heterogeneity in radioactive contamination (Møller & Mousseau, 2015) around study locations (black dotes) in Ukraine and Belarus: Bobor (1:51.154017, 29.536783), Voronkov (2:50.205691, 30.830013), Pripyat (3:51.412333, 30.040132), Dytiatki (4:51.11288, 30.150321), Neglyubka 1 (5:52.765012, 31.537877), Neglyubka 2 (6:52.750472, 31.514022), Kolbovka (7:52.779136, 31.473382), Bolsuny (8:53.064516, 31.295611), Dubovy Log (9:52.533333, 31.366667), Berezki (10:52.558227, 31.52736), Pisky (11:51.08939, 30.047221), Rudnia (12:51.28901, 29.783602) and Vesniane (13:51.30655, 29.630955)

Here, we tested if exposure to radioactive contamination correlated with body temperature of wild barn swallows breeding in the Chernobyl area. We hypothesized that exposure to increased radioactive contamination would impair normal thermoregulation in birds. On the one hand, exposure to high contamination could lead to decreased body temperature, similar to responses observed for acute doses of toxic chemicals (Bicego et al., 2007; Gordon, 2018; Leon, 2008). Alternatively, animals chronically exposed to radioactive contaminants could suffer up‐regulation of body temperature (i.e., fever). Individual physiological status and phenology can influence individual exposure to contaminants and are known to correlate with barn swallow body temperature (Møller, 2010). Thus, we tested how these factors, including body condition, behavior (risk of capture, tonic immobility), social environment (colony size), age, and phenology (time and date of measures), influenced the relation between radioactive contamination and body temperature. We expected that young and healthy individuals to be more resistant to harmful compounds in the environment (Møller et al., 2005; Rudolph et al., 1999). We also expected that variation among individuals in activity level could affect food consumption rates and hence ingestion rates of contaminants (Auer et al., 2018; Sol et al., 2018) with consequent effects on body temperature. Phenology could be important as barn swallows overwinter outside of the Chernobyl area (in Africa), and therefore, their exposure to contamination would begin at the time of their arrival in the spring (De Vocht et al., 2015). To test these hypotheses, we analyzed a very large dataset (1,246 records) collected over 12 years for 13 colonies of wild barn swallows, *Hirundo rustica*, in an area encompassing low, medium, and highly contaminated regions of Ukraine and Belarus (Figure 1; Bonisoli‐Alquati et al., 2010).

## 2 MATERIAL AND METHODS

### 2.1 Study area and colonies

Research on barn swallows (*Hirundo rustica* Linnaeus, 1758) in the Chernobyl area was conducted between 2008 and 2019 for this study (Figure 1). The study area consisted of abandoned farmland, including a mixture of fallow fields, pastures, and forests (Bonisoli‐Alquati et al., 2010; Møller et al., 2005, 2011). Thirteen study sites, distanced at least 2.2 km from each other, were located across a radioactive contamination gradient in central northern Ukraine and south‐eastern Belarus (Figure 1). The study was conducted during the breeding season of barn swallows as this species does not overwinter in the region and thus is only exposed to radiation between late April and early August. Barn swallows in the areas breed inside barns and other buildings. Most of the colonies were abandoned by birds during the study due to closures of doors to buildings or destruction of buildings resulting in only a small number of sites being occupied at the end of the research period (Ellegren et al., 1997; Møller et al., 2006). Birds were captured at study colonies with mist nets and were individually marked with rings at first capture. The number of individuals in each colony, the year of survey, and colony size were recorded, along with the date and time of capture.

### 2.2 Environmental contamination

Environmental radioactive contamination among study sites varied by a factor 425, from 0.02 to 8.50 (μGy/h) with a mean of 0.81 (standard deviation = 2.19). The level of ambient radioactive contamination of the capture sites was measured at ground level using a hand‐held dosimeter (Model: Inspector, SE International, Inc., Summertown, TN, USA as described in Mousseau & Møller, 2013). These measurements of radioactive contamination were highly repeatable for measurements collected during the same day, within season and among years (intraclass correlation coefficients >0.89, *p* < .0001; Galván et al., 2014). The field measurements were cross‐validated against an updated large dataset of deposition observations of ^137^Cs over Europe, from the public Radioactivity Environmental Monitoring database of the European Union Joint Research Center [Figure 1; Evangeliou et al., 2016; linear regression between averages of field measurements and averages of 10 closest dataset records located <2.5 km from the study sites: *β* = 0.61 (0.21), *z* = 2.91, *p* = .0036, Pearson's product‐moment correlation: *r* = .70, *t* = 2.56, *df* = 7, *p* = .037].

### 2.3 Individual variation

Animal core body temperature was measured at time of capture, with a TA804C thermometer equipped with a thermocouple probe, by inserting a 1.0 cm long tip of the probe into the cloaca for around 60 s. This is a highly repeatable measure among recaptured individuals (intraclass correlation coefficient = 0.74, *p* < .0001; Møller, 2010; and weakly correlated with radioactive contamination: Figure S1). Birds were subject to morphological measurement, including bill, keel, and tarsus lengths with a digital caliper (to the nearest 0.1 mm) and body mass with a Pesola spring balance (with a precision of 0.1 g; Møller, 2010). Individuals were assigned an age of one year when first captured in a colony (Møller, 1994). Barn swallows that had once been captured in a given colony never moved to another farm (in the Chernobyl region), with another population at Kraghede, Denmark only showing breeding dispersal to a maximum distance of 750 m (Møller, 1994). The order of capture of individuals, within the colony and year, was recorded to estimate individual risk‐taking behavior, potentially affecting their exposure to predation (Møller, 2010; Stuber et al., 2013). Individual tonic immobility behavior was estimated at the time of release of birds following measurements. Tonic immobility is a measure of fearfulness estimated by placing the bird on its back and recording the time that it took for the bird to right itself and fly away (Boissy, 1995; Forkman et al., 2007; Hoagland, 1928; Jones, 1986; Møller & Ibáñez‐Álamo, 2012). All handling of birds during measurements was performed by the same researcher (APM) to avoid observer bias.

### 2.4 Statistical analyses

All variables were log_10_ transformed, centered, and scaled prior to analyses in order to facilitate comparison of regression coefficients among traits. Principal component analysis (with varimax rotation) based on the correlation matrix was used to statistically partition variation in bill, tarsus, and keel lengths (mm), and body mass (g) into principal components. The analysis showed that variance in bill (standardized loading = 0.76), tarsus (0.73), and keel length (0.39), but not in body mass (0.04), were allocated to the first component (Figure S2). This component can be interpreted as structural size (explaining 32% of the variation in morphological traits). Variance in body mass (0.97), but not in bill (0.15), tarsus (−0.23), or keel length (0.06), was allocated to the second component, that can be interpreted as body condition of animals (explaining 25% of variation in traits; Tables S1–S3).

Single multivariate mixed regression analysis was used to estimate the relationship between radioactive contamination and core body temperature. To investigate whether individual and phenological variables (body size and condition, risk of capture, tonic immobility, age, colony size, and time and date of measure) influenced that relationship, two‐way interactions between radioactive contamination and the individual and phenological variables were tested. Nonlinear effects of the predictors on body temperature were also tested by inclusion of their quadratic terms. Random effects of individual ID, year, and site of study were accounted for in the model. The model was simplified by step‐wise backward elimination of weak two‐way interactions and quadratic terms, while insignificant linear terms were retained in the final test. In cases where interaction terms were detected, additional analyses were performed. Additional analyses tested the direction of relationships between body temperature and radioactive contamination on data constrained to the highest and the lowest 10% of records of the interacting predictor. Body temperature and radioactive contamination did not differ between males and females, and sex did not affect relationships between temperature and radiation, thus sex was omitted from subsequent analyses. All estimates (*β*) are reported “±” standard errors. Analyses were performed with the “glmmADMB” R package (Fournier et al., 2012; Skaug et al., 2016).

## 3 RESULTS

### 3.1 Descriptive statistics

In total, 1,246 records from 1,091 barn swallows were collected (129 birds were repeatedly measured 2–6 times). Core body temperature measurements varied from 37.3 to 43.7℃, with a mean (and standard deviation) of 41.13℃ (1.10). There was substantial variation in structural size and body mass among individuals (Table S1), that was partitioned into effects of body size (PC1, loaded by lengths of bill, keel, and tarsus) and body condition (PC2, loaded by body mass) with principal component analysis (Figure S2).

### 3.2 Body condition, radioactive contamination, and body temperature

**FIGURE 2:**
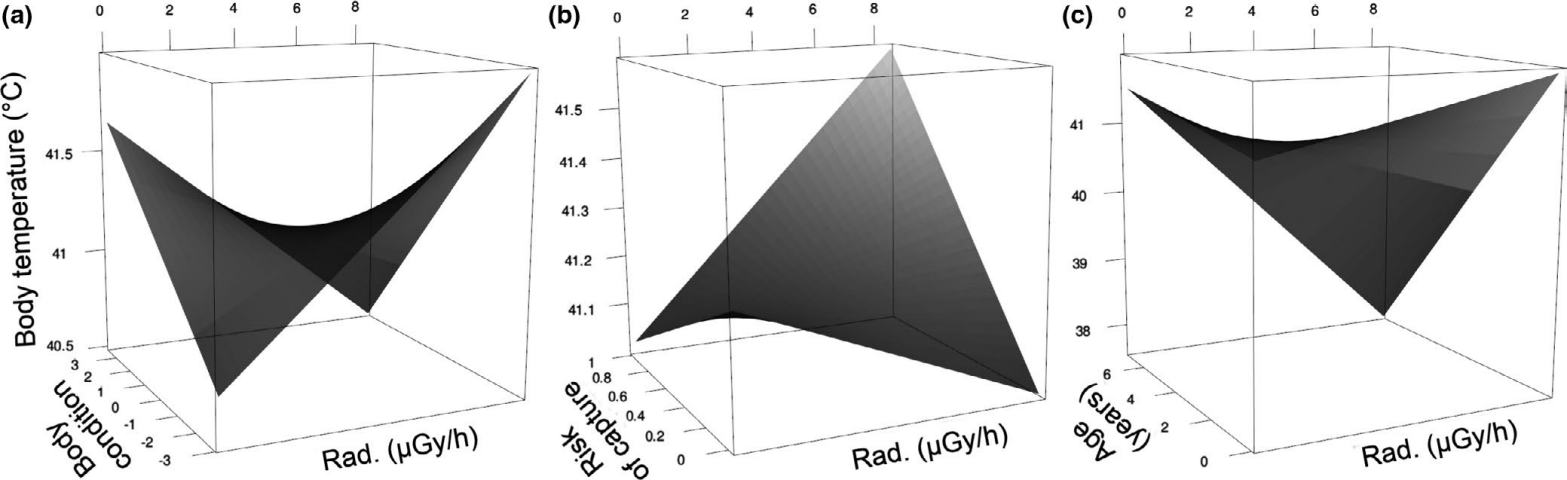
The effect of radioactive contamination level (Rad.), when interacting with body condition (a), risk of capture (b) and age of individuals (c), on core body temperature (predicted values) of wild barn swallows in the Chernobyl area. Predicted values were derived from analysis on log_10_ transformed, scaled, and centered variables (Table 1)

Multivariate regression analysis (including body size and condition, risk of capture, tonic immobility, age, colony size, and time and date of measures) revealed interactions with radioactive contamination affecting body temperature (Table 1). First, the analysis showed that radioactive contamination interacted with body condition to affect body temperature (*β* = −0.09 ± 0.03, *z* = −3.51, *p* = .0004; Figure 2a). Body temperature increased with increasing radioactive contamination in birds with low body condition (*N* = 123, *β* = 0.46 ± 0.11, *z* = 4.20, *p* < .0001), but not in birds with high body condition (*N* = 116, *β* = 0.04 ± 0.11, *z* = 0.36, *p* = .72).

**TABLE 1:** Relation between core body temperature in barn swallows and environmental radioactive contamination in Chernobyl Exclusion Zone

	*β* (*SE*)	*z*	*p*
Intercept	−0.06 (0.256)	−0.25	.80
Body size	0.02 (0.025)	0.90	.37
Body condition	0.10 (0.023)	4.33	<.0001
Radioactive contamination (RC)	0.03 (0.051)	0.65	.51
Risk of capture	−0.04 (0.033)	−1.14	.25
Tonic immobility	−0.07 (0.022)	−3.23	.0012
Age	0.01 (0.025)	0.01	.99
Colony size	0.26 (0.047)	5.55	<.0001
Time	0.12 (0.037)	3.16	.0016
Date	0.44 (0.043)	10.28	<.0001
Body size^2^	0.04 (0.016)	2.26	.024
Body condition^2^	−0.03 (0.015)	−2.08	.037
RC * Body condition	−0.09 (0.025)	−3.51	.0004
RC * Risk of capture	0.06 (0.025)	2.26	.024
RC * Age	−0.10 (0.048)	−2.14	.033
RC * Time	0.17 (0.033)	5.16	<.0001
RC * Date	−0.10 (0.025)	−3.93	<.0001

Note

Results from multivariate mixed regression analysis testing variation predictors: radiation, body size and body condition (derived from principal component analyses), risk of capture, tonic immobility, age, colony size and time and date of body temperature measures. Variance (standard deviations) of random effects: individual (*N* = 1,091) = 0.04 (0.04), year (*N* = 12) = 0.34 (0.15), and site (*N* = 13) = 0.32 (0.15). vif index for predictors <1.45. Weak quadratic terms of radioactive contamination and colony size (*p* > .04) were removed (Table S4).

### 3.3 Risk of capture, radioactive contamination, and body temperature

Second, the same multivariate analysis (Table 1) showed that radioactive contamination interacted with risk of capture to affect body temperature (*β* = 0.06 ± 0.03, *z* = 2.26, *p* = .024; Figure 2b). Body temperature increased with increasing radiation in individuals with high risk of capture (*N* = 125, *β* = 0.15 ± 0.07, *z* = 2.17, *p* = .030), but not in birds with low risk (*N* = 129, *β* = 0.14 ± 0.08, *z* = 1.73, *p* = .084). In the latter analysis, interaction between time and radiation was significant (Table S5).

### 3.4 Age, radioactive contamination, and body temperature

Third, the same multivariate analysis (Table 1) revealed that radioactive contamination interacted with age to affect body temperature (*β* = −0.10 ± 0.05, *z* = −2.14, *p* = .033; Figure 2c). The effect was weak and insignificant for young (≤1 year: *N* = 993, *β* = 0.06 ± 0.05, *z* = 1.23, *p* = .22) and old birds (≥2 year: *N* = 129, *β* = −0.80 ± 0.52, *z* = −1.55, *p* = .12; Table S6).

### 3.5 Time and date of measurements, radioactive contamination, and body temperature

**FIGURE 3:**
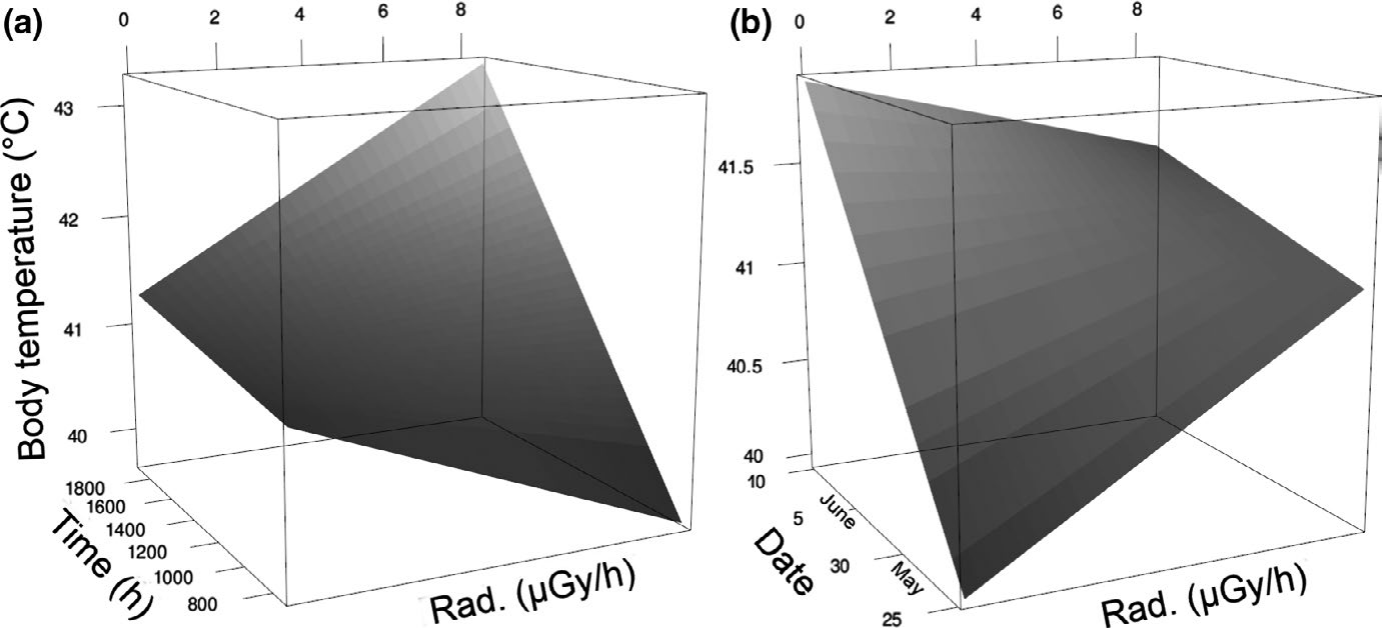
The effect of radioactive contamination level (Rad.), when interacting with time (a) and date of capture (b), on core body temperature (predicted values) of wild barn swallows in the Chernobyl area. Predicted values were derived from analysis on log_10_ transformed, scaled, and centered variables (Table 1)

Fourth, the multivariate analysis (Table 1) also revealed that radioactive contamination interacted with time of day (*β* = 0.17 ± 0.03, *z* = 5.16) and date of measurements (*β* = −0.10 ± 0.03, *z* = −3.93, *p* < .0001) to affect body temperature (Figure 3). Body temperature correlated with radiation in birds measured in the evening (after 16:00: *N* = 185, *β* = 0.41 ± 0.14, *z* = 2.88, *p* = .004), but not in the morning (before 10:00: *N* = 46, *β* = −0.80 ± 0.51, *z* = −1.55, *p* = .12; Table S7). Body temperature correlated with radiation in birds measured during spring (25–28 May: *N* = 159, *β* = 0.40 ± 0.08, *z* = 5.28, *p* < .0001), but not in birds measured during summer (8–10 June: *N* = 136, *β* = −0.01 ± 0.17, *z* = −0.01, *p* = .99).

### 3.6 Tonic immobility, colony size, body size and body condition, and body temperature

**FIGURE 4:**
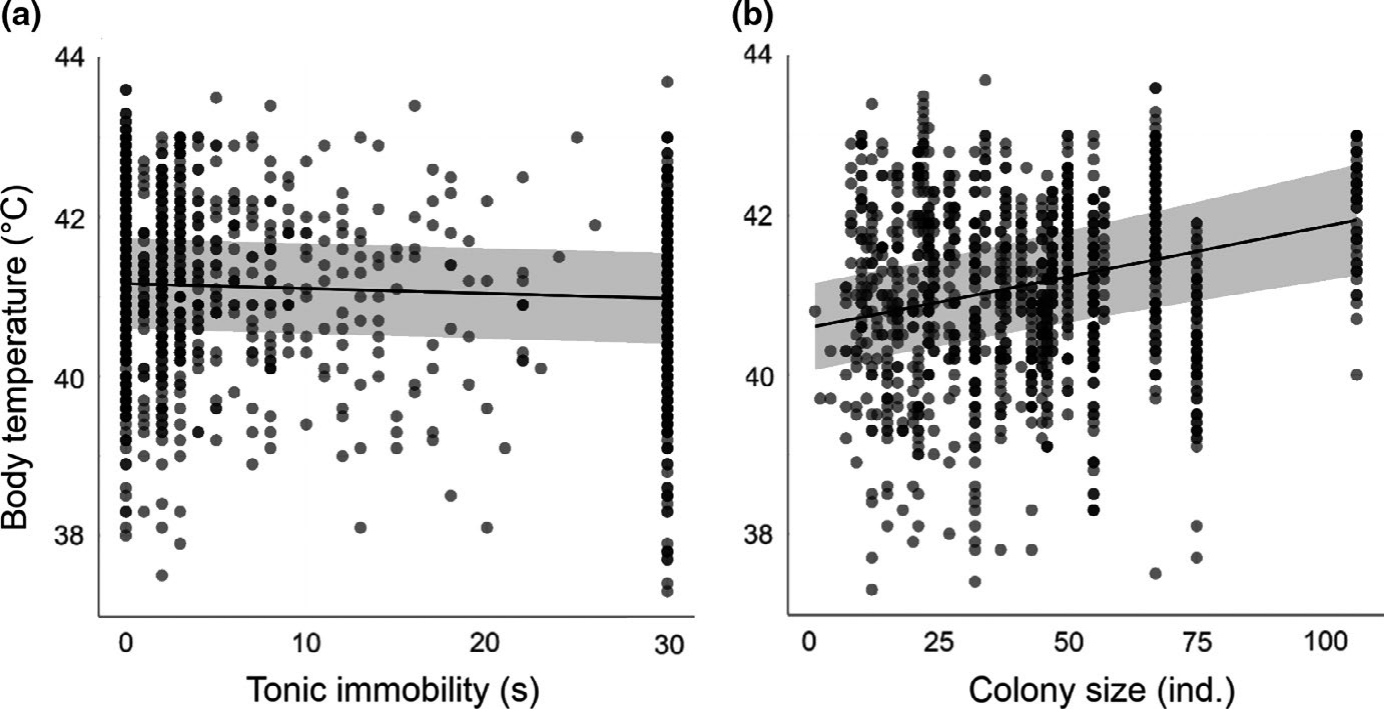
The effects (and their confidence intervals) of tonic immobility (a) and colony size (b) on body temperature (predicted values) of wild barn swallows in the Chernobyl area. Predicted values were derived from analysis on log_10_ transformed, scaled, and centered variables (Table 1)

**FIGURE 5:**
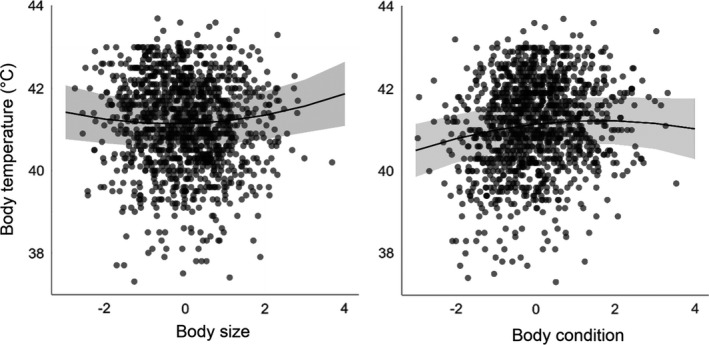
The quadratic effects (and their confidence intervals) of body size (a; PC1) and body condition (b; PC2) on body temperature (predicted values) of wild barn swallows from the Chernobyl area, as derived from regression analysis on log_10_ transformed, scaled, and centered variables (Table 1)

Finally, the same multivariate analysis (Table 1) revealed that body temperature negatively correlated with tonic immobility (*β* = −0.07 ± 0.02, *z* = −3.23, *p* = .0012) and positively with colony size (*β* = 0.26 ± 0.05, *z* = 5.55, *p* < .0001; Figure 4). Quadratic terms of opposite signs for body size (*β* = 0.04 ± 0.02, *z* = 2.26, *p* = .024) and body condition (*β* = −0.03 ± 0.01, *z* = −2.08, *p* = .037) also predicted variation in body temperature (Figure 5).

## 4 DISCUSSION

Environmentally induced down‐ or up‐regulation of body temperature can compromise energetic balance and fitness of individuals. Here, we have demonstrated that barn swallow body temperature was affected by the level of exposure to radioactive contamination released from the Chernobyl accident and that this effect was modulated by individual quality and phenology. Our results showed that radioactive exposure can influence body temperature and that there are complex mechanisms that determine the magnitude and direction of these effects. Body temperature radiosensitivity was affected by body condition, risk of capture, and time and date of capture. Birds of low body condition and high risk of capture that were measured late during the day but early during the season showed increased radiosensitivity and expressed higher core body temperatures proportional to the contamination level.

Birds of poorer body condition (lighter), but not structurally small birds, showed significantly higher radiosensitivity than birds with high body condition (heavier). Light animals more often expressed up‐regulation of temperature (i.e., fever) when exposed to increased radioactive contamination. Body condition is often related to health, quality, or vigor (Beauchamp et al., 2020; Labocha et al., 2014; Peig & Green, 2010). Our results showed that animals of relatively poor condition were more prone to be affected by contamination and develop fever more often. While in previous work body temperature increased significantly with body condition (Møller, 2010), here we showed that condition dependency of body temperature can be hampered by radioactive contamination. Thus, our data suggest that radioactive exposure disrupts physiological processes of thermoregulation in barn swallows, but also that this depends on body condition of birds. Whether this pattern emerges from direct detrimental effects of ingested radionuclides on individual physiology or from indirect effects of increased infection level on weakened host organisms (i.e., immune response) remains to be investigated. Recent studies indeed showed that radioactive exposure constrains natural variation in wild animal gut microbiota (Czirják et al., 2010; Lavrinienko et al., 2020, 2021). Those results, together with observations that energy metabolism and immunosuppression pathways are affected in contaminated areas in Chernobyl (Kesäniemi, Jernfors, et al., 2019; Kesäniemi, Lavrinienko, et al., 2019), suggest that mechanisms impairing temperature regulation in radioactive exposed organisms can be diverse.

In addition to body condition, some, but not all individual and phenological factors, mediated the effect of radioactive contamination on body temperature. Specifically, while tonic immobility and colony size were not significant interaction terms, individual risk of capture, time and day of capture, and age at capture mediated this relationship. Opposing effects of radiation on body temperature between age classes were relatively weak and insignificant when tested separately for young and old birds. This was surprising considering that young and healthy individuals generally perform better in terms of thermoregulation, while thermal homeostasis in old animals is often prone to disruption (Blatteis, 2012; Sanchez‐Alavez et al., 2011; Shibasaki et al., 2013).

Body temperature correlated with radioactive exposure among individuals with high risks of capture but not for difficult to capture birds. Variation among individuals in terms of ease of capture is widely observed among species and populations (Biro & Dingemanse, 2009; Simons et al., 2015). Risk‐taking or bolder animals can be more prone to capture, and the risk of capture can be related to locomotory activity; animals with increased activity in their daily area are detected easier (Stuber et al., 2013). High levels of activity require greater energetic support (Boratyński, 2020; Boratyński et al., 2020) that can be related to increase foraging effort, thus ingesting more radionuclides with contaminated food and water. Based on our observations, it can be hypothesized that bolder and more active animals are more exposed to contamination in their habitat.

Body temperature positively correlated with radioactive contamination in animals captured in the evening, but not in birds investigated during the morning. One explanation might be that exposure to contamination accumulates during daily activity of birds and that the physiological responses increase over the course of the day in response to accumulated radionuclides (De Vocht et al., 2015). Body temperature also increased with increasing radiation in birds measured during spring, but not in animals measured during summer. The mechanisms underlying these relationships are not at all understood but one could speculate that increased intensity of foraging immediately following migration in the spring, combined with the added energetic demands of reproduction, could influence exposure to contamination thus affecting physiology and thermoregulatory ability. Experimental approaches will be necessary to resolve mechanisms of such temporal trends. It can be hypothesized that acute and chronic effects are overlapping in our data, due to cumulative exposure over a long period of time. For instance, animals from highly contaminated areas could have suffered effects similar to those observed in animals exposed to acute doses of toxic chemicals (such as alcohol, benzene, paraldehyde, DDT, among others; Bicego et al., 2007; Leon, 2008), and down‐regulate their body temperature. However, selective foraging of birds from very contaminated colonies in clean locations, where food resources are more abundant in Chernobyl, could also influence that result (Møller & Mousseau, 2009).

## 5 CONCLUSIONS

Understanding the physiological effects of anthropogenic stressors (e.g., radioactive contamination) that lead to changes in fitness of organisms is an important issue given increasing pollution of natural habitats (Lourenço et al., 2016). To the best of our knowledge, this study is the first investigation of how radioactive contamination affects wild animal body temperature. Our results suggest that temperature regulation in birds can be affected by radioactive contamination in the environment, interacting with individual quality and phenology, and that birds often developed fever in more radioactive habitats. These results highlight complex mechanisms of how thermal biology is impacted by increased radioactive exposure and is modulated by animal condition, behavior, and phenology. Temperature regulation is a natural mechanism employed by many organisms in response to, for example, variation in climatic condition or pathogen infection, and the expression of temperature and fever and its consequences may vary among populations (Adelman et al., 2010; Bastos et al., 2021; Elliot et al., 2002). A variety of processes related to temperature regulation may be affected in radioactive areas leading to diverse mechanisms for adaptation to local contamination. Such processes might include mechanisms related to heat generation and dissipation, performance capacity determining animal mobility and migration, and maintenance physiology determining energetic processes supporting basic life functions (Pettersen et al., 2018). Investigation of these processes, along with detailed phenological study, will be crucial for understanding mechanisms governing radionuclides, and other pollutants, circulation within biological systems, and the risks related to them. Warming of environmental temperatures will expose animals to increased risk of overheating, that, together with environmental contamination, can exaggerate organisms' well‐being whenever environmental contaminants lead to body temperature up‐regulation and fever.

## CONFLICT OF INTEREST

We declare no conflict of interest.

## AUTHOR CONTRIBUTIONS

**Zbyszek Boratyński:** Formal analysis (lead); writing‐original draft (lead). **Timothy A. Mousseau:** Data curation (equal); funding acquisition (equal); investigation (equal); resources (equal); writing‐review & editing (equal). **Anders Pape Møller:** Conceptualization (equal); data curation (equal); formal analysis (equal); funding acquisition (equal); investigation (equal); methodology (equal); writing‐review & editing (equal).

## Supporting information

Appendix S1

## Data Availability

Data for this study are available at Figshare public repository: https://doi.org/10.6084/m9.figshare.14610369.v1.
