# Supplementary material for: Individual quality and phenology mediate the effect of radioactive contamination on body temperature in Chernobyl barn swallows
Source: Ecol Evol. 2021 Jun 2;11(13):9039–48. doi: 10.1002/ece3.7742 (PMC8258232; doi:10.1002/ece3.7742)
Supplement: Supplementary file 1 — Appendix S1 [file ECE3-11-9039-s001.doc]

Supplementary material to manuscript (Evolutionary Applications):

**The effect of radioactive contamination on body temperature in Chernobyl barn swallows**

**
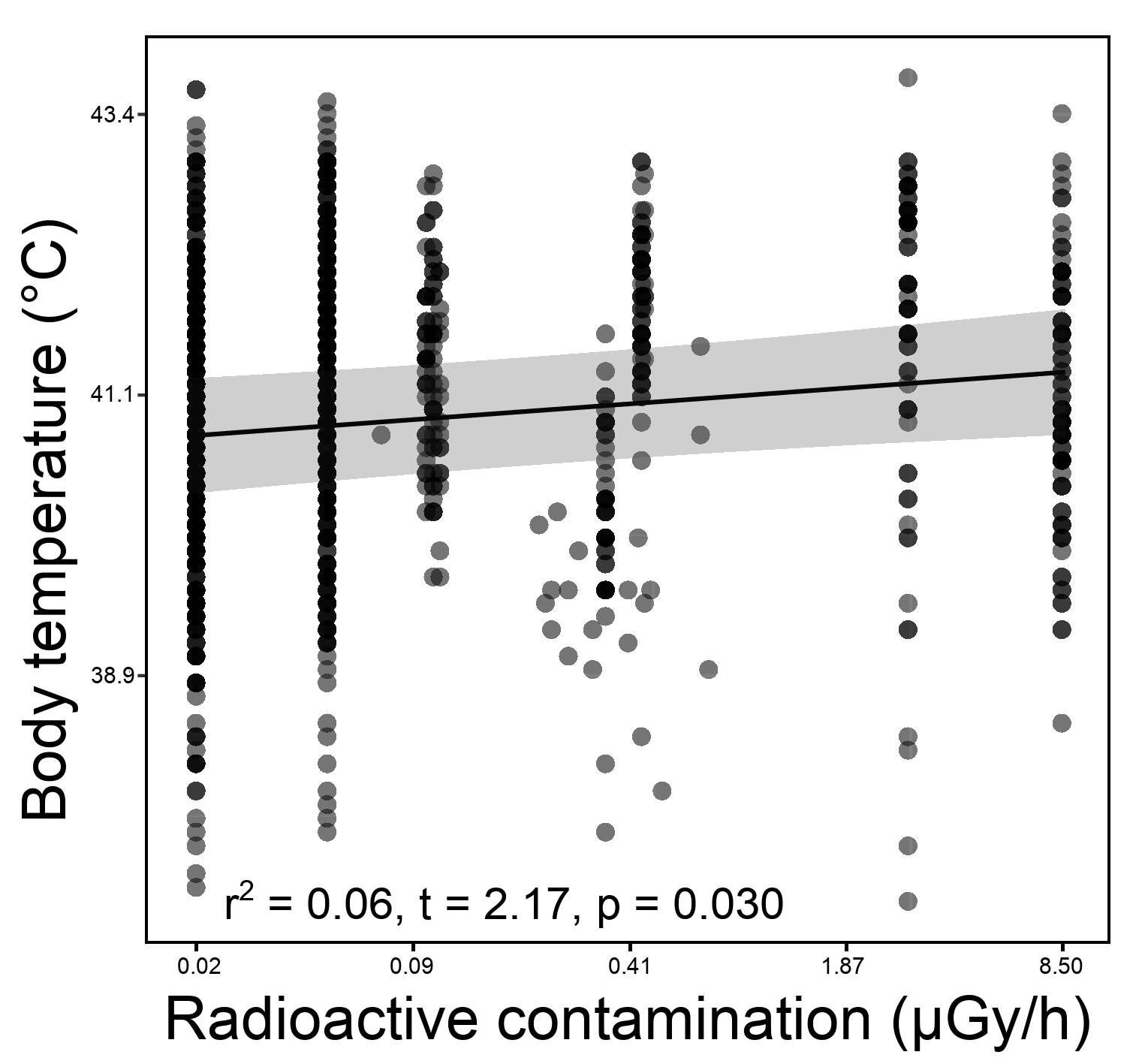
**

Figure S1. Relation (regression line and its confidence intervals, gray shading) between core body temperature (predicted values) of barn swallows and environmental radioactive contamination in Chernobyl Exclusion Zone (presented on log-log scale). Predicted values were derived from model including only body size and body condition as covariates and random factors of animal ID, and year and site of study. r^2^ represents Pearson's product-moment correlation between residual body temperature, accounting for variation in body size and condition, and variance among individuals, years and sites, and environmental radioactive contamination level.

Table S1. Variation it traits measured in barn swallows from Chernobyl area.

|  | *mean (SD)* | *range* |
| --- | --- | --- |
| Body size (PC1) | -2E-17 (1.00) | -2.77-3.68 |
| Body condition (RC2) | 2E-17 (1.00) | -2.95-3.54 |
| Bill length (mm) | 8.23 (0.60) | 6.07-11.12 |
| Tarsus length (mm) | 11.22 (0.75) | 9.01-13.99 |
| Keel length (mm) | 21.11 (0.88) | 17.39-29.68 |
| Body mass (g) | 18.81 (1.45) | 15.0-24.0 |
| Body temperature (°C) | 41.13 (1.10) | 37.3-43.7 |
| Environmental radiation (μGy/h) | 0.81 (2.19) | 0.02-8.50 |
| Risk of capture (order) | 0.49 (0.30) | 0.0-1.0 |
| Tonic immobility (s) | 11.60 (12.93) | 0-30 |
| Age (year) | 1.23 (0.64) | 0-7 |
| Colony size (individuals) | 43.26 (22.74) | 1-106 |
| Time of capture (hh:mm) | 12:57 (2:37) | 7:15-19:06 |
| Day of capture (since 1 May) | 33.53 (4.09) | 25-40 |

Principal component analysis

Variables were scaled and centered prior to analyses. Varimax rotation was applied to calculate two rotated principal components.

Figure S2. Morphological division to structural body size (PC1) and physiological body condition (PC2) dimensions, as explained by body mass and bill, tarsus and keel lengths.

Table S2. Variance explained by rotated principal components.

|  |  | *PC1* | *PC2* |
| --- | --- | --- | --- |
| SS | loadings | 1.26 | 1.02 |
| Proportion | variance | 0.32 | 0.25 |
| Proportion | explained | 0.55 | 0.45 |

Table S3. Loading for rotated principal components.

|  | *PC1* | *PC2* | *h2* | *u2* | *com* |
| --- | --- | --- | --- | --- | --- |
| Bill length | **0.77** | 0.15 | 0.61 | 0.39 | 1.1 |
| Tarsus length | **0.73** | -0.23 | 0.58 | 0.42 | 1.2 |
| Keel length | **0.39** | 0.06 | 0.15 | 0.85 | 1.0 |
| Body mass | 0.04 | **0.97** | 0.94 | 0.06 | 1.0 |

Table S4. Results from multivariate mixed regression analysis explaining variation in body temperature among barn swallows in the Chernobyl area, before removal of weak (p > 0.04) quadratic terms (Table 1). vif, the variance inflation factor.

|  | *β* | *s.e.* | *z* | *p* | *vif* |
| --- | --- | --- | --- | --- | --- |
| Intercept | -0.04 | 0.25 | -0.17 | 0.87 |  |
| Body size | 0.02 | 0.02 | 0.83 | 0.41 | 1.3 |
| Body condition | 0.10 | 0.02 | 4.46 | 0.00001 | 1.1 |
| Radiation | 0.39 | 0.19 | 2.03 | 0.043 | 2.1 |
| Risk of capture | 0.00 | 0.04 | 0.09 | 0.93 | 1.0 |
| Tonic immobility | -0.07 | 0.02 | -3.30 | 0.0010 | 1.1 |
| Age | 0.00 | 0.02 | -0.20 | 0.84 | 1.1 |
| Colony size | 0.22 | 0.05 | 3.99 | 0.00007 | 1.5 |
| Time | 0.16 | 0.04 | 3.96 | 0.0001 | 1.5 |
| Date | 0.46 | 0.04 | 10.43 | <2E-016 | 1.5 |
| Body size^2^ | 0.03 | 0.02 | 2.16 | 0.031 | 1.0 |
| Body condition^2^ | -0.03 | 0.02 | -2.14 | 0.033 | 1.0 |
| Radiation^2^ | -0.10 | 0.05 | -2.04 | 0.041 | 1.7 |
| Colony size^2^ | 0.05 | 0.03 | 2.04 | 0.042 | 1.3 |
| Radiation*Body size | 0.05 | 0.02 | 2.13 | 0.033 | 1.0 |
| Radiation*Body condition | -0.10 | 0.02 | -3.89 | 0.0001 | 1.0 |
| Radiation*Risk | -0.06 | 0.03 | -2.44 | 0.015 | 1.0 |
| Radiation*Age | -0.12 | 0.05 | -2.48 | 0.013 | 1.0 |
| Radiation*Time | 0.17 | 0.03 | 4.94 | 0.00000 | 1.1 |
| Radiation*Date | -0.09 | 0.03 | -3.59 | 0.0003 | 1.0 |

Variance (standard deviations) of random effects: individual (N = 1091) = 0.04 (0.04), year (N = 12) = 0.33 (0.15), and site (N = 13) = 0.25 (0.13).

Table S5. Results from two multivariate mixed regression analyses explaining variation in body temperature among barn swallows in the Chernobyl area. Data constrained to animals with low risk of capture (10% of minimum distribution). Upper panel presents results with significant interaction and lower after excluding it from the model.

|  | *β* | *s.e.* | *z* | *p* |
| --- | --- | --- | --- | --- |
| Intercept | 0.17 | 0.222 | 0.77 | 0.44 |
| Radiation | 0.00 | 0.098 | -0.04 | 0.97 |
| Tonic immobility | -0.18 | 0.075 | -2.44 | 0.015 |
| Colony size | 0.21 | 0.077 | 2.76 | 0.0058 |
| Time | -0.13 | 0.088 | -1.53 | 0.13 |
| Radiation*Time | 0.19 | 0.083 | 2.30 | 0.022 |
|  |  |  |  |  |
|  | *β* | *s.e.* | *z* | *p* |
| Intercept | 0.04 | 0.201 | 0.18 | 0.86 |
| Radiation | 0.14 | 0.078 | 1.73 | 0.084 |
| Tonic immobility | -0.15 | 0.075 | -1.94 | 0.052 |
| Colony size | 0.20 | 0.079 | 2.51 | 0.012 |

Table S6. Results from four multivariate mixed regression analyses explaining variation in body temperature among barn swallows in the Chernobyl area. Upper panels present results with significant interactions and lower after excluding them from the models.

|  | birds younger them 1 year | | | |  | birds older them 3 years | | | |
| --- | --- | --- | --- | --- | --- | --- | --- | --- | --- |
|  | *β* | *s.e.* | *z* | *p* |  | *β* | *s.e.* | *z* | *p* |
| Intercept | -0.04 | 0.256 | -0.14 | 0.89 |  | 0.37 | 0.409 | 0.91 | 0.36 |
| Body size | -0.02 | 0.028 | -0.75 | 0.45 |  | - | - | - | - |
| Body condition | 0.10 | 0.025 | 4.21 | <0.0001 |  | -0.58 | 0.287 | -2.03 | 0.042 |
| Radiation | 0.06 | 0.047 | 1.23 | 0.22 |  | -0.01 | 0.595 | -0.01 | 0.99 |
| Risk of capture | -0.05 | 0.037 | -1.25 | 0.21 |  | - | - | - | - |
| Tonic immobility | -0.07 | 0.025 | -2.66 | 0.0078 |  | - | - | - | - |
| Colony size | 0.28 | 0.054 | 5.27 | <0.0001 |  | - | - | - | - |
| Time | 0.10 | 0.043 | 2.42 | 0.015 |  | - | - | - | - |
| Date | 0.45 | 0.048 | 9.41 | <0.0001 |  | 0.74 | 0.172 | 4.32 | <0.0001 |
| Body size^2^ | 0.06 | 0.018 | 3.48 | 0.0005 |  | - | - | - | - |
| Body condition^2^ | -0.04 | 0.017 | -2.28 | 0.022 |  | - | - | - | - |
| Radiation*Body condition | -0.07 | 0.025 | -2.99 | 0.0028 |  | -1.97 | 0.802 | -2.45 | 0.014 |
| Radiation*Risk | 0.06 | 0.026 | 2.14 | 0.032 |  | - | - | - | - |
| Radiation*Time | 0.17 | 0.034 | 4.89 | <0.0001 |  | - | - | - | - |
| Radiation*Date | -0.10 | 0.026 | -4.08 | <0.0001 |  | - | - | - | - |
|  |  |  |  |  |  |  |  |  |  |
|  | *β* | *s.e.* | *z* | *p* |  | *β* | *s.e.* | *z* | *p* |
| Intercept | 0.03 | 0.242 | 0.13 | 0.90 |  | 0.15 | 0.394 | 0.37 | 0.71 |
| Body size | -0.03 | 0.028 | -1.14 | 0.26 |  | - | - | - | - |
| Body condition | 0.10 | 0.025 | 3.99 | <0.0001 |  | - | - | - | - |
| Radiation | 0.06 | 0.047 | 1.25 | 0.21 |  | -0.80 | 0.512 | -1.55 | 0.12 |
| Colony size | 0.30 | 0.053 | 5.71 | <0.0001 |  | - | - | - | - |
| Time | 0.15 | 0.027 | 5.63 | <0.0001 |  | - | - | - | - |
| Date | 0.41 | 0.048 | 8.69 | <0.0001 |  | 0.64 | 0.176 | 3.62 | 0.00029 |
| Body size^2^ | 0.06 | 0.018 | 3.15 | 0.0016 |  | - | - | - | - |
| Body condition^2^ | -0.05 | 0.017 | -2.64 | 0.0082 |  | - | - | - | - |

Table S7. Results from four multivariate mixed regression analyses explaining variation in body temperature among barn swallows in the Chernobyl area. Upper panels present results with significant interactions and lower after excluding them from the models.

|  | birds measured in the morning | | | |  | birds measured in the evening | | | |
| --- | --- | --- | --- | --- | --- | --- | --- | --- | --- |
|  | *β* | *s.e.* | *z* | *p* |  | *β* | *s.e.* | *z* | *p* |
| Intercept | 0.37 | 0.409 | 0.91 | 0.36 |  | -0.39 | 0.438 | -0.90 | 0.37 |
| Body condition | -0.58 | 0.287 | -2.03 | 0.042 |  | 0.12 | 0.050 | 2.29 | 0.022 |
| Radiation | -0.01 | 0.595 | -0.01 | 0.99 |  | 0.46 | 0.153 | 3.02 | 0.0026 |
| Risk of capture | - | - | - | - |  | -0.14 | 0.067 | -2.04 | 0.041 |
| Age | - | - | - | - |  | 0.01 | 0.037 | 0.15 | 0.88 |
| Date | 0.74 | 0.172 | 4.32 | 0.00002 |  | 0.59 | 0.098 | 5.99 | <0.0001 |
| Body condition^2^ | - | - | - | - |  | -0.11 | 0.032 | -3.44 | 0.00057 |
| Radiation*Age | - | - | - | - |  | -0.16 | 0.060 | -2.61 | 0.0092 |
| Radiation*Risk | -1.97 | 0.802 | -2.45 | 0.014 |  | 0.21 | 0.056 | 3.72 | 0.00020 |
|  |  |  |  |  |  |  |  |  |  |
|  | *β* | *s.e.* | *z* | *p* |  | *β* | *s.e.* | *z* | *p* |
| Intercept | 0.15 | 0.394 | 0.37 | 0.71 |  | -0.21 | 0.401 | -0.53 | 0.60 |
| Body condition | - | - | - | - |  | 0.13 | 0.052 | 2.57 | 0.010 |
| Radiation | -0.80 | 0.512 | -1.55 | 0.12 |  | 0.41 | 0.141 | 2.88 | 0.0040 |
| Date | 0.64 | 0.176 | 3.62 | 0.0003 |  | 0.49 | 0.098 | 5.02 | <0.0001 |
| Body condition^2^ | - | - | - | - |  | -0.12 | 0.034 | -3.34 | 0.00085 |

**R codes used in analysis**

data<- read.table("dataR.csv", header=TRUE)

library(psych)

library(rptR)

library(glmmADMB)

############select variables for PCA

varPCA <- c("Bill_L", "R_Tars", "Keel", "B_Mass")

dataPCA <- data[varPCA]

names(dataPCA)

dataPCAscal <- scale(dataPCA, center=TRUE, scale=TRUE)

##########PCA

PCA_mod <- principal(dataPCAscal, nfactors=2, scores=TRUE, rotate = "varimax")

PCA_mod

summary(PCA_mod)

PCA_scores <- as.data.frame(PCA_mod$scores)

############merging data

dataPA <- cbind(data, PCA_scores)

attach(dataPA)

names(dataPA)

###########################INTERACTIONS

dataPA$sT_C <- scale(dataPA$T_C, center=TRUE, scale=TRUE)

dataPA$sKeel <- scale(dataPA$Keel, center=TRUE, scale=TRUE)

dataPA$sBill <- scale(dataPA$Bill_length, center=TRUE, scale=TRUE)

dataPA$sTars <- scale(dataPA$R_Tars, center=TRUE, scale=TRUE)

dataPA$sMass <- scale(dataPA$B_Mass, center=TRUE, scale=TRUE)

dataPA$sRad <- scale(dataPA$Radiation, center=TRUE, scale=TRUE)

dataPA$sTime <- scale(dataPA$Time, center=TRUE, scale=TRUE)

dataPA$sDate <- scale(dataPA$Date, center=TRUE, scale=TRUE)

dataPA$sRoC <- scale(dataPA$risk_of_capture, center=TRUE, scale=TRUE)

dataPA$sCols <- scale(dataPA$Col_size, center=TRUE, scale=TRUE)

dataPA$sFeather_mites <- scale(dataPA$Feather_mites, center=TRUE, scale=TRUE)

dataPA$sTonic <- scale(dataPA$Tonic_immobility, center=TRUE, scale=TRUE)

dataPA$sAge <- scale(dataPA$Age, center=TRUE, scale=TRUE)

dataPA$Year <- as.factor(dataPA$Year)

dataPA$Site <- as.factor(dataPA$Site)

dataPA$IndID <- as.factor(dataPA$IndID)

###########################################Full reduction

mod <- lmer(sT_C ~ sRad*sTime + sRad*sDate + sRad*sTonic + sRad*sFeather_mites + sRad*sAge + sRad*sCols + sRad*sRoC + sRad*RC1 + sRad*RC2 + I(RC1^2) + I(RC2^2) + I(sRad^2) + I(sTime^2) + I(sDate^2) + I(sTonic^2) + I(sAge^2) + I(sCols^2) + I(sRoC^2) + I(sFeather_mites^2) + (1|IndID) + (1|Year) + (1|Site), data = dataPA)

summary(mod)

vif(mod)

modd <- glmmadmb(sT_C ~ TLD*sRad + RC1 + RC2 + sRad + sRoC + sTonic + sAge + sCols + sTime + sRad*RC1 + sRad*RC2 + sRad*sRoC + sRad*sAge + sRad*sTime + sRad*sDate + I(RC1^2) + I(RC2^2) + I(sRad^2) + I(sCols^2) + (1|IndID) + (1|Year) + (1|Site), data = dataPA, family = "gaussian")

summary(modd)

vif(modd)

modd$sd_S

##########################reduction of p > 0.040

mod <- lmer(sT_C ~ sRad*sTime + sRad*sDate + sTonic + sRad*sAge + sCols + sRad*sRoC + RC1 + sRad*RC2 + I(RC1^2) + I(RC2^2) + (1|IndID) + (1|Year) + (1|Site), data = dataPA)

summary(mod)

vif(mod)

modd <- glmmadmb(sT_C ~ TLD + RC1 + RC2 + sRad + sRoC + sTonic + sAge + sCols + sTime + sRad*RC2 + sRad*sRoC + sRad*sAge + sRad*sTime + sRad*sDate + I(RC1^2) + I(RC2^2) + (1|IndID) + (1|Year) + (1|Site), data = dataPA, family = "gaussian")

summary(modd)

vif(modd)

modd$sd_S
